# Activation of Nrf2/HO-1 Pathway and Human Atherosclerotic Plaque Vulnerability: An In Vitro and In Vivo Study

**DOI:** 10.3390/cells8040356

**Published:** 2019-04-16

**Authors:** Susanna Fiorelli, Benedetta Porro, Nicola Cosentino, Alessandro Di Minno, Chiara Maria Manega, Franco Fabbiocchi, Giampaolo Niccoli, Francesco Fracassi, Simone Barbieri, Giancarlo Marenzi, Filippo Crea, Viviana Cavalca, Elena Tremoli, Sonia Eligini

**Affiliations:** 1 Centro Cardiologico Monzino, I.R.C.C.S., 20138 Milan, Italy; susanna.fiorelli@cardiologicomonzino.it (S.F.); benedetta.porro@ccfm.it (B.P.); nicola.cosentino@cardiologicomonzino.it (N.C.); alessandro.diminno@cardiologicomonzino.it (A.D.M.); chiara.manega@cardiologicomonzino.it (C.M.M.); franco.fabbiocchi@cardiologicomonzino.it (F.F.); simone.barbieri@cardiologicomonzino.it (S.B.); giancarlo.marenzi@cardiologicomonzino.it (G.M.); elena.tremoli@cardiologicomonzino.it (E.T.); sonia.eligini@cardiologicomonzino.it (S.E.); 2 Department of Cardiovascular & Thoracic Sciences, Fondazione Policlinico Universitario A. Gemelli, I.R.C.C.S., Università Cattolica del Sacro Cuore, 00168 Rome, Italy; gniccoli73@hotmail.it (G.N.); francesco.fracassi@yahoo.it (F.F.); filippo.crea@rm.unicatt.it (F.C.)

**Keywords:** oxidative stress, nuclear factor erythroid 2–related factor 2, heme-oxygenase-1, macrophages, plaque vulnerability, optical coherence tomography

## Abstract

Reactive oxygen species (ROS) induce nuclear factor erythroid 2–related factor 2 (Nrf2) activation as an adaptive defense mechanism, determining the synthesis of antioxidant molecules, including heme-oxygenase-1 (HO-1). HO-1 protects cells against oxidative injury, degrading free heme and inhibiting ROS production. HO-1 is highly expressed in macrophages during plaque growth. Macrophages are morpho-functionally heterogeneous, and the prevalence of a specific phenotype may influence the plaque fate. This heterogeneity has also been observed in monocyte-derived macrophages (MDMs), a model of macrophages infiltrating tissue. The study aims to assess oxidative stress status and Nrf2/HO-1 axis in MDM morphotypes obtained from healthy subjects and coronary artery disease (CAD) patients, in relation to coronary plaque features evaluated in vivo by optical coherence tomography (OCT). We found that MDMs of healthy subjects exhibited a lower oxidative stress status, lower Nrf2 and HO-1 levels as compared to CAD patients. High HO-1 levels in MDMs were associated with the presence of a higher macrophage content, a thinner fibrous cap, and a ruptured plaque with thrombus formation, detected by OCT analysis. These findings suggest the presence of a relationship between in vivo plaque characteristics and in vitro MDM profile, and may help to identify patients with rupture-prone coronary plaque.

## 1. Introduction

The progression of coronary atherosclerotic plaque and its destabilization with plaque rupture and thrombus formation are the key mechanisms of acute myocardial infarction (AMI) [1,2]. Post-mortem reports have demonstrated that the vulnerable plaque is characterized by a large lipid/necrotic core, a thin fibrous cap, and a great amount of resident macrophages [3]. Macrophages are versatile cells and, in relation to microenvironmental stimuli, they respond by activating different signal transduction pathways, expressing several receptors, and acquiring specific phenotypes. At coronary atherosclerotic plaque level, macrophage population is also characterized by morphological and functional heterogeneity that may enhance plaque growth and/or rupture [4].

An increasing body of evidence suggests that oxidative stress is closely associated with the atherosclerotic process and plaque instability [5,6] through different pathological mechanisms, including endothelial dysfunction, lipid oxidation, expression of adhesion molecules, and monocyte recruitment [7,8,9]. In response to oxidative stress stimuli, cells implement several defense mechanisms and, among them, the activation of nuclear erythroid factor 2 – related factor 2 (Nrf2)/heme oxygenase-1 (HO-1) pathway was reported to be associated with atherosclerosis [10,11]. Under unstressed conditions, Nrf2 is constitutively expressed and sequestrated in the cytoplasm by Keap1 (Kelch-like erythroid cell-derived protein with cap ’n’ collar homology-associated protein 1), inducing its proteasomal degradation. On the other hand, under an oxidative stress stimulus, the complex Keap1/Nrf2 dissociates itself, and Nrf2 translocates into the nucleus. At this level, Nrf2 binds the antioxidant responsive element and promotes the transcription of proteins with antioxidant activity. Among them, HO-1 plays a fundamental role in the antioxidant mechanism within the cell by degrading the prooxidant heme to carbon monoxide, biliverdin, and ferrous ion [11]. In addition, HO-1 induction partially inhibits nicotinamide adenine dinucleotide phosphate (NADPH) oxidase activity representing a mechanism of cytoprotection against oxidative stress [12].

HO-1 expression is induced by various proatherogenic stimuli and risk factors for cardiovascular diseases [13,14,15]. HO-1 is highly expressed in atherosclerotic plaques, mainly localized in macrophages and foam cells [16], where its antioxidant and anti-inflammatory properties could be fundamental to counteract the development of early stage lesions [17]. In particular, HO-1 reduces the immune cell recruitment and infiltration [18], regulates the macrophage polarization also driving a phenotypic shift towards an anti-inflammatory phenotype [19,20], and inhibits the maturation of dendritic cells [21], thus affecting lesion formation.

HO-1 may also affect plaque progression and its anti-atherogenic role was highlighted in in vitro and in vivo models. Indeed, the induction of HO-1 in co-cultures of human aortic endothelial cells and smooth muscle cells inhibited the monocyte oxidized low density lipoprotein (oxLDL)-dependent chemotaxis [22]. Accordingly, the evidence of an augmented atherogenesis after HO-1 inhibition and an attenuation in the development of atherosclerotic lesion after HO-1 induction confirms this protective role. Indeed, accelerated and more advanced atherosclerotic plaques were described in HO-1 knockout mice [23]. Moreover, the inhibition of HO-1 expression in hyperlipidemic rabbits [24] or in LDL-receptor deficient mice fed with high-fat diet [25] resulted in greater atherosclerotic lesions and increased plasma and tissue lipid peroxide levels.

Despite these preclinical studies, HO-1 levels, higher than those of healthy subjects, were observed in lymphocytes and monocytes isolated from coronary artery disease (CAD) patients. In particular, its expression was higher in patients with AMI than in those with stable angina (SA) [26]. More recently, Cheng et al. showed an increased HO-1 expression in carotid atherosclerotic lesions with a vulnerable phenotype. Of note, the HO-1 levels positively correlated with plaque macrophage and lipid content, and they inversely correlated with stable plaque features, like the presence of intra-plaque smooth muscle cells and collagen [16]. Overall, animal and human studies suggest that HO-1 reflects the severity of atherosclerosis, indicating that a high level of this protein in vulnerable plaque macrophages may represent an antioxidant response, aiming at counteracting the oxidative damage inside atherosclerotic plaque [16,27].

Since resident macrophages are not easily obtainable and manageable, macrophages obtained from a spontaneous differentiation of monocytes (MDMs) are considered to be a good in vitro model to study tissue macrophages. We have previously reported the co-existence of two main and different macrophage morphotypes (round and spindle cells) after 7-day culture of human monocytes isolated from healthy subjects [28]. Similarly to tissue macrophages heterogeneity [4], different MDM morphotypes showed different functional properties: in particular, round-shaped cells were reminiscent of M2 macrophage phenotype with anti-inflammatory and reparative characteristics. On the contrary, spindle-shaped cells showed a pro-inflammatory profile resembling M1 macrophages [28].

In our more recent study, we demonstrated that the peculiar morpho-phenotype profile of MDMs isolated from CAD patients is associated with the characteristics of coronary vulnerable plaque, as assessed by optical coherence tomography (OCT) [29]. This accurate intracoronary imaging technique allows the visualization and the characterization of the atherosclerotic plaque [30,31] providing its detailed architecture and highlighting the rupture prone plaques.

Currently, no data have been provided on the association between HO-1 levels and macrophage phenotype in CAD patients. In this work, we investigated HO-1 levels and the activation of Nrf2/HO-1 axis in different MDM morphotypes obtained from healthy subjects and CAD patients, also in relation to coronary plaque morphology and activity, as analyzed in vivo by OCT.

## 2. Materials and Methods

### 2.1. Study Population

Thirty consecutive CAD patients undergoing coronary angiography, due to SA or AMI, as their first manifestation of ischemic heart disease, were enrolled at Centro Cardiologico Monzino, Milan, Italy. SA was defined as angina on effort with a stable pattern of symptoms for at least the last six months prior to admission; AMI diagnosis encompassed patients presenting with non-ST-elevation (NSTEMI) or ST-elevation-acute myocardial infarction (STEMI). NSTEMI was defined as chest pain at rest in the last 48 h preceding the admission associated with evidence of transient ST-segment depression on 12-lead electrocardiogram and normal (unstable angina) or elevated (NSTEMI) serum troponin I (TnI) levels. The diagnosis of STEMI was based on typical symptoms lasting more than 30 min and new ST-segment elevation at the J point in ≥2 contiguous leads. Exclusion criteria were: Previous history of CAD; severe chronic heart failure, severe heart valve disease, acute and chronic infections, liver diseases, neoplasia, evidence of immunologic disorders, recent (<3 months) surgical procedures or trauma and use of anti-inflammatory or immunosuppressive drugs and antioxidant supplements. All AMI included patients underwent percutaneous coronary intervention, specifically within 24 h from admission for NSTEMI and within 12 h of symptom onset for STEMI patients. OCT assessment was performed both in SA and in AMI in order to investigate coronary plaque features. The control group consisted of 10 healthy subjects, with neither history of CAD, nor cardiovascular risk factors, nor inflammatory disorders, and specifically not taking any cardiovascular therapy. The ethics committee approved the study protocol, and all participants provided written informed consent to participate in the study. The study was performed according to the Declaration of Helsinki.

### 2.2. Monocyte Isolation and Culture

Venous blood samples were drawn from the antecubital vein of healthy subjects and CAD patients when fasting into tubes containing ethylenediaminetetraacetic acid (EDTA) (9.3 mM; Vacutainer Systems, Becton Dickinson, USA). Mononuclear cells were isolated by Ficoll-Paque Plus (GE Healthcare, Milan, Italy) density centrifugation and plated (2 × 10^6^/mL) in 35 mm well plates (Primaria, Falcon, Como, Italy) as previously described [11]. After 90 min, non-adherent cells were removed and adherent cells were cultured over 7 days at 37 °C (5% CO2) in Medium 199 (Lonza, Milan, Italy) supplemented with 2 mM l-glutamine, 100 U/mL penicillin, 100 µg/mL streptomycin, and 10% autologous serum without replacement of the medium throughout the entire culture period. MDM morphology was examined by phase contrast microscopy (Axiovert 200 M; Zeiss, Milan, Italy) at 20× or 40× magnification. MDMs were defined spindle/elongated when a length > 70 µm and a width < 30 µm were detected, and round MDMs when width and length were similar and >30 to 40 µm. Cells, whose morphology and dimension did not satisfy the above criteria, were classified as undefined.

### 2.3. Liquid Chromatography Tandem Mass Spectrometry (LC-MS/MS) Analysis

For the determination of reduced (GSH) and oxidized (GSSG) glutathione levels, MDMs were washed with phosphate buffer saline (PBS) and detached by gentle scraping. After centrifugation (400× *g*, 10 min), the supernatant was removed and MDMs were lysed in PBS containing 0.1 µg of leupeptin, 0.2 M benzamidine, and 1 µg of trypsin inhibitor. Lysed cells were mixed in a 1:1 (*v*/*v*) ratio with 10% trichloroacetic acid (TCA) containing 1 mM EDTA. After centrifugation (10,000× *g*, 10 min) at room temperature (RT), the supernatant was diluted 1:20 with 0.1% formic acid. The analysis was performed using LC-MS/MS method as previously described [32]. Liquid chromatography was performed on Luna analytical PFP column (100 × 2.0 mm, 3 µm) using an Accela HPLC pump system (Thermo Fisher Scientific, Milan, Italy). Mass spectrometric analysis was performed using a TSQ Quantum Access (Thermo Fisher Scientific) triple quadrupole mass spectrometer coupled with electrospray ionization (ESI) operated in positive mode.

### 2.4. Western Blot Analysis

Western blot analysis was performed on MDMs total lysate, and on MDMs cytosolic and nuclear fractions.

To obtain MDMs total lysate, cells were harvested and lysated in a buffer composed by 20 mM Tris, 4% sodium dodecyl sulfate (SDS) and 20% glycerol, containing 1 mM sodium orthovanadate, 1 mM NaF, 1 µg/mL leupeptin hemisulfate, 1 mM benzamidine hydrochloride, 1 mM EDTA, 10 µg/mL soybean trypsin inhibitor, 0.5 mM pefabloc, 0.5 mM dithiothreitol (DTT) [33].

For the isolation of cytosolic fraction, MDMs were harvested in 50 µL of buffer containing 10 mM HEPES, pH 7.9, 1.5 mM MgCl_2_, 10 mM KCl, 0.5 mM DTT and then Triton X-100 (0.2% final concentration) was added. Cells were centrifuged at 15,000× *g* for 1 min at 4 °C to separate cytosols from nuclei [34]. The supernatant containing cytosolic fraction was used for the analysis and the pellet was used for nuclear fraction extraction.

The pellet nuclear fraction was washed with a buffer (20 mM HEPES, pH 7.9, 400 mM NaCl, 25% glycerol, 1.5 mM MgCl_2_, 0.2 mM EDTA) containing 0.5 mM PMSF, and 0.5 mM DTT and centrifuged at 15,000× *g* (10 min, 4 °C) [34]. The supernatant containing nuclear fraction was used for the analysis.

SDS-polyacrylamide gel electrophoresis (PAGE) was performed as previously described [33]. After blotting, membranes carrying MDMs total lysates were incubated overnight at 4 °C with primary antibodies directed against HO-1 (1:250) (catalogue number: ab13248; Abcam, Milan, Italy) or Nrf2 (1:200) (Santa Cruz Biotechnology; catalogue number: sc-722, Milan, Italy).

Membranes carrying MDMs cytosolic and nuclear fraction samples were incubated overnight at 4 °C with primary antibodies directed against Nrf2 (1:200) (Santa Cruz Biotechnology). After incubation with horseradish peroxidase-conjugated anti-mouse (1:10,000, catalogue number: 715-035-151) or anti-rabbit secondary antibody (1:5000, catalogue number: 111-035-003) (Jackson ImmunoResearch Labs Inc., Li StarFISH, Milan, Italy), as appropriate, for 1 h at RT, protein bands were detected by chemiluminescence. β-Actin was used as internal standard for control of protein load.

### 2.5. Immunofluorescence Analysis

Immunofluorescence was performed as previously described [35]. Fixed MDMs were incubated overnight at 4 °C with a monoclonal rabbit anti-human HO-1 antibody (1:100) (Abcam), or with a polyclonal rabbit anti-human Nrf2 antibody (1:200) (Santa Cruz Biotechnology). Detection was performed with Alexa Fluor 488 (1:200, catalogue number: A11034, 60 min at RT) (Life Technologies Italia, Monza, Italy). Nuclei were visualized by Hoechst 33258 (1:10,000, catalogue number: B2883) (Sigma-Aldrich, Milan, Italy). Negative control experiments were performed by omitting the primary antibodies. Fluorescence quantification was performed as previously described [33]. Data are expressed as mean ± SD of fluorescence intensity/µm^2^ for each MDM morphotype, subtracted of the negative control value obtained in the absence of primary antibody. Multiple fields of view (at least three fields, 400× magnification) were captured for each culture.

### 2.6. OCT Image Acquisition and Analysis

OCT examination was performed in AMI patients at culprit lesion and in SA patients at the minimal lumen area (MLA) site. Some plaque characteristics were determined such as the measurement of the thickness of fibrous cap, lipid content, and macrophage accumulation. Fibrous cap thickness was defined as the minimum distance from the coronary artery lumen to the inner border of the lipid pool and a thin-cap fibroatheroma (TCFA) was defined as a minimal fibrous cap thickness ≤ 65 µm; thick-cap fibroatheroma was a plaque with a minimal fibrous cap thickness > 65 μm. The max lipid arc was measured on the frame with the largest lipid core by visual screening. A plaque showing two or more lipid containing quadrants was considered a lipid-rich plaque. A lipid plaque with fibrous cap discontinuity and cavity formation inside the plaque was defined as rupture plaque. A thrombus was defined as an irregular mass protruding into the lumen with a measured dimension > 250 μm.

The macrophage infiltration (MØI) in the analyzed lesions by OCT, has been assessed as previously reported [36], according to the International Working Group for Intravascular Optical Coherence Tomography (IWG-IVOCT) Consensus standards [37]. Briefly, macrophages were qualitatively identified on raw OCT data within a 300 × 125 μm^2^ (lateral x axial) region of interest (ROI). In particular, macrophages have been visualized by OCT imaging as signal-rich, distinct, or confluent punctate regions that exceed the intensity of background speckle noise and generate a backward shadowing. For caps having a thickness < 125 μm^2^, the depth of the ROI was matched to the cap thickness. Median filtering was performed with a 3 × 3 square kernel to remove speckle noise. In plaques with MØI, quantitative evaluation of macrophage content was obtained by measuring the normalized standard deviation (NSD) known to have a high degree of positive correlation with histological measurements of macrophage content, by using a dedicated software provided by S. Jude medical [38,39]. In particular, NSD was measured for each pixel within each cap using a 125 μm^2^ window centered at the pixel location:

NSD (x,y) = [σ (x,y)125 μm^2^/(Smax-Smin)] × 100 (1)

where NSD (x,y) is the normalized standard deviation of the OCT signal at pixel location (x,y), Smax is the maximum OCT image value, and Smin is the minimum OCT image value. Pixels within the (125 × 125) μm^2^ window that did not overlap with the segmented cap were excluded.

### 2.7. Statistical Analysis

Continuous variables are presented as mean ± SD, variables not normally distributed are presented as median and interquartile ranges (IQR), and categorical variables as absolute numbers and percentages. Comparisons between ‘healthy subjects’ vs. ‘CAD patients’ groups were performed using independent samples *t*-Test for normally distributed variables and Wilcoxon rank-sum test for not normally distributed variables.

Comparisons between ‘healthy subjects’ vs. ‘SA’, ‘NSTEMI’, and ‘STEMI’ groups were performed using ANOVA Test for normally distributed variables and Kruskal–Wallis Test for not normally distributed variables. Categorical variables were compared using Fisher’s exact test. Post-hoc testing of main effects was performed using Bonferroni adjustment for multiple comparisons (α/[number of comparisons]). Correlations between variables were determined using Spearman’s rank test. Trends of variation from healthy subjects to STEMI patients were assessed by general linear models. All tests were two-sided, and a *p* value of less than 0.05 was required for statistical significance. All calculations were computed by using SAS software package v9.4 (SAS Institute Inc., Cary, NC, USA).

## 3. Results

### 3.1. Clinical Features

Demographic and clinical characteristics of the enrolled subjects are shown in Table 1. CAD patients had higher body mass index (BMI) and they were more frequently males. Moreover, as expected, in CAD group there was a prevalence of subjects with cardiovascular risk factors as smoking, diabetes, dyslipidemia, hypertension, and family history of cardiovascular disease. Despite there being more subjects with dyslipidemia among CAD patients, the LDL cholesterol values were similar between patients and healthy subjects, as the result of pharmacological treatment. Furthermore, CAD patients had higher levels of glycaemia and of C-reactive protein (hs-CRP), an inflammatory marker.

Of the 30 consecutive CAD patients, 10 (33.3%) had a diagnosis of SA, whereas 20 (66.6%) of AMI (10 NSTEMI (33.3%) and 10 STEMI (33.3%)). Among CAD patients, those with STEMI showed a higher BMI, higher levels of hs-CRP, creatinine, glycaemia, total cholesterol, triglycerides, TnI, and creatine phosphokinase-MB (CK-MB). In addition, lower high-density lipoprotein (HDL) cholesterol levels were observed in AMI patients. No difference in admission therapy was observed among CAD patients.

### 3.2. Oxidative Stress Status

**Figure 1:**
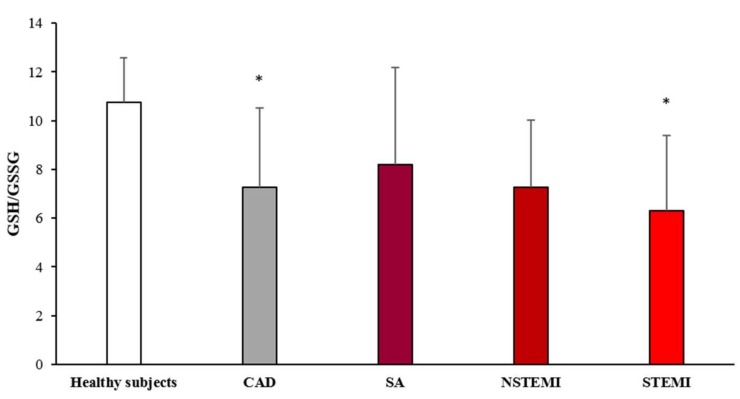
GSH/GSSG evaluation in monocyte-derived macrophages (MDMs). MDMs were obtained from CAD patients and healthy subjects. Data are expressed as mean ± SD and derive from independent cultures obtained from 10 healthy subjects and 30 CAD patients (SA *n* = 10; NSTEMI *n* = 10; STEMI *n* = 10. * *p* < 0.05 vs. healthy subjects.

The levels of GSH and GSSG, whose ratio is a recognized index of oxidative stress, were measured in MDMs obtained from the study population. The results are showed in Figure 1. The GSH/GSSG ratio was significantly lower in CAD patients as compared to healthy subjects. The analysis of the different clinical presentations of CAD revealed a progressive decrease of GSH/GSSG ratio in MDMs going from SA, NSTEMI, to STEMI patients (*p*_trend_ < 0.005).

### 3.3. HO-1 and Nrf2 Expression

**Figure 2:**
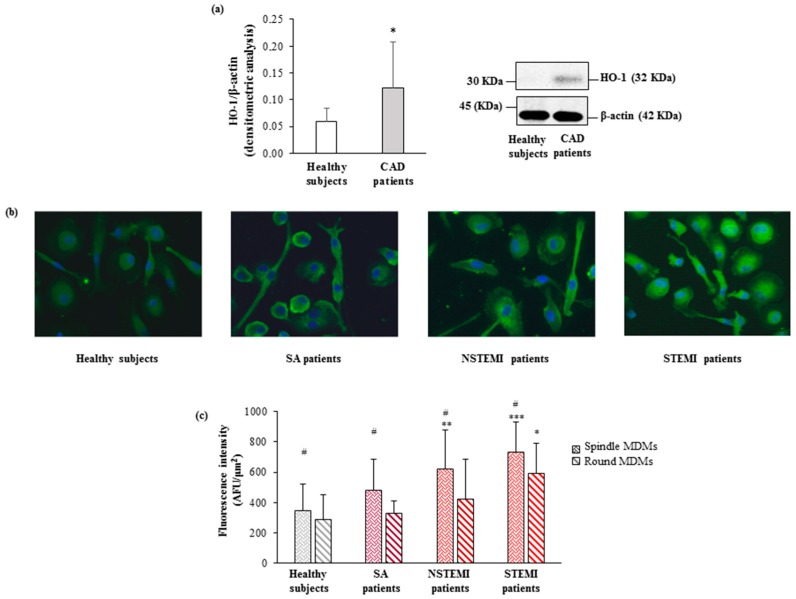
HO-1 levels in CAD patients and healthy subjects. (**a**) The protein levels of HO-1 were detected by western blot analysis. β-actin was used as a control of protein loading. Densitometry is shown in the bar graph. Data are expressed as mean ± SD and derive from MDMs obtained from 10 healthy subjects and 17 CAD patients. (**b**) Representative images of HO-1 in round and spindle MDMs obtained from healthy subjects and CAD patients (400× original magnification), nuclei were visualized by Hoechst 33258. (**c**) Quantitative analysis of HO-1 in round and spindle MDMs. Data are expressed as mean ± SD of fluorescence intensity/µm^2^ (at least three fields, 400× magnification, were analyzed) and data derive from independent cultures obtained from 10 healthy subjects and 30 CAD patients (SA *n* = 10; NSTEMI *n* = 10; STEMI *n* = 10. ^#^ *p* < 0.05 vs. round; * *p* < 0.05, ** *p* < 0.01, *** *p* < 0.001 vs. healthy subjects.

MDMs of CAD patients displayed higher levels of HO-1 protein as compared to those observed in healthy subjects (0.12 ± 0.09 vs. 0.06 ± 0.02, *p* < 0.05) (Figure 2a). Moreover, the immunofluorescence analysis showed higher protein levels in spindle compared to round cells in all study patients’ groups. (Figure 2b) with a significant increase in both MDM morphotypes of STEMI patients. A progressive increase was also shown going from healthy subjects to STEMI patients (*p*_trend_ round < 0.0002, *p*_trend_ spindle < 0.0001; Figure 2c).

**Figure 3:**
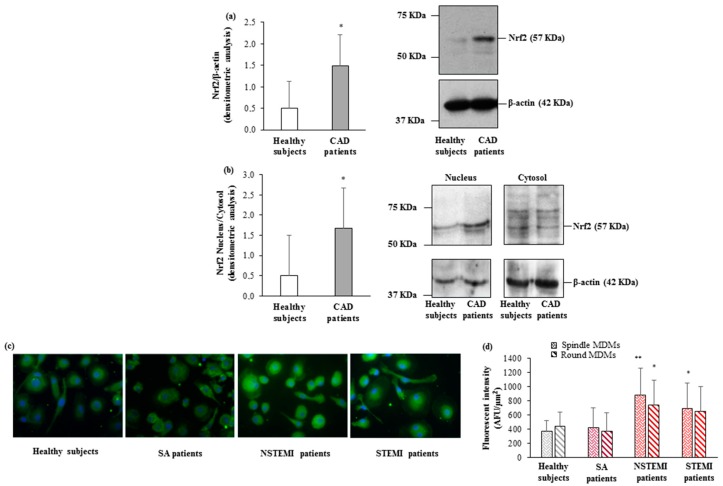
Nrf2 levels in CAD patients and healthy subjects. (**a**,**b**) Nrf2 levels in (**a**) total cellular lysate, (**b**) nuclear and cytosolic compartments were detected by western blot analysis. β-actin was used as a control of protein loading. Densitometry is shown in the bar graph. Data are expressed as mean ± SD and derive (**a**) from MDMs obtained from 10 healthy subjects and 17 CAD patients; (**b**) from MDMs obtained from 5 healthy subjects and 5 CAD patients. (**c**) Representative images of Nrf2 in round and spindle MDMs obtained from healthy subjects and CAD patients (400× original magnification), nuclei were visualized by Hoechst 33258. (**d**) Quantitative analysis of Nrf2 in round and spindle MDMs. Data are expressed as mean ± SD of fluorescence intensity/µm^2^ (at least three fields, 400× magnification, were analyzed) and derive from independent cultures obtained from 10 healthy subjects and 30 CAD patients (SA *n* = 10; NSTEMI *n* = 10; STEMI *n* = 10). * *p* < 0.05, ** *p* < 0.01, vs. healthy subjects.

This behavior was mirrored by the levels of the transcription factor Nrf2. Higher levels of total Nrf2 were detected in CAD patients as compared to healthy subjects (1.49 ± 0.73 vs. 0.51 ± 0.62, *p* < 0.01) (Figure 3a). In addition, the evaluation of Nrf2 levels in cytoplasmic and nuclear fractions demonstrated a significant increase of Nrf2 translocation into the nucleus in CAD patients as compared to those of healthy subjects (Figure 3b). The immunofluorescence analysis of the MDM morphotypes evidenced significantly higher Nrf2 protein levels in both spindle and round MDMs of AMI patients (NSTEMI and STEMI) as compared to those of healthy subjects (Figure 3c). Furthermore, an increasing trend in protein levels was detected in both MDM morphotypes paralleling the severity of the clinical presentations (*p*_trend_ round < 0.02, *p*_trend_ spindle = 0.06) (Figure 3d).

### 3.4. Association Between In Vivo Plaque Morphology and HO-1 Levels in MDMs

The coronary plaque characteristics in CAD patients are illustrated in Table 2.

**Figure 4:**
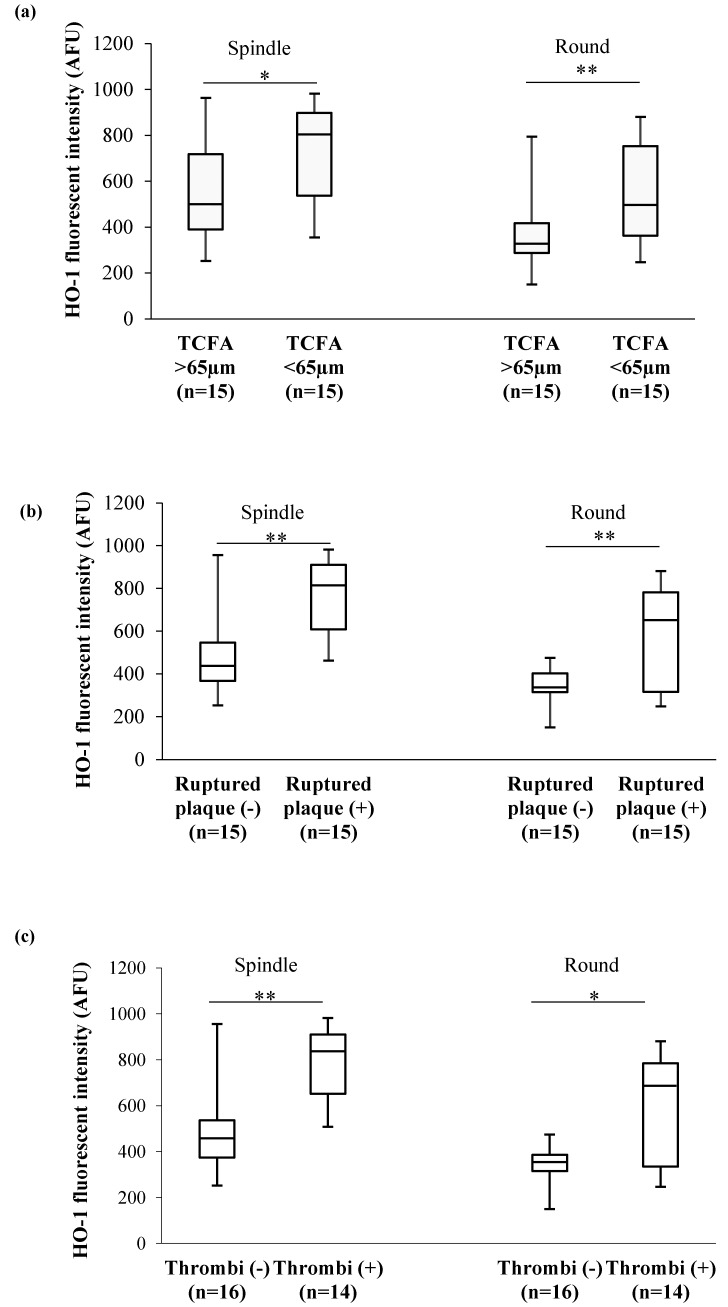
In vivo plaque features and HO-1 levels in MDM morphotypes. (**a**) Association between HO-1 levels and thin cap fibroatheroma (TCFA), (**b**) fibrous cap integrity, (**c**) presence of thrombi detected by means of optical coherence tomography (OCT). Data are expressed as median and IQR. * *p* < 0.05, ** *p* < 0.01.

Patients with high levels of HO-1 in both MDM morphotypes more frequently displayed a TCFA (*p* = 0.049 and *p* = 0.015, spindle and round, respectively) (Figure 4a), a ruptured plaque (*p* = 0.001 and *p* = 0.036, spindle and round, respectively) (Figure 4b), and presence of thrombi (*p* = 0.0005 and *p* = 0.028, spindle and round, respectively) (Figure 4c).

**Figure 5:**
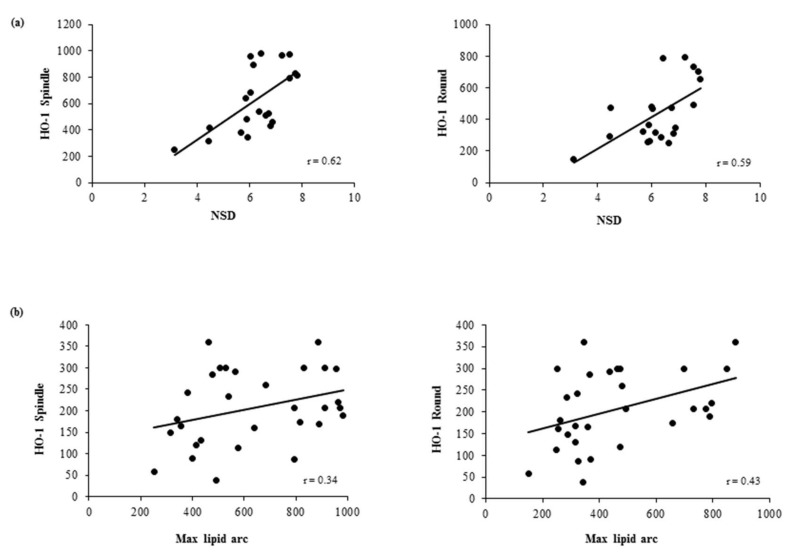
Correlations between in vivo plaque features and HO-1 levels in MDM morphotypes. Correlation between HO-1 levels in round and spindle MDMs and (**a**) macrophage content (NSD) and (**b**) max lipid arc, detected by means of OCT in in vivo plaque.

In addition, in both MDM morphotypes we observed significant positive correlations between HO-1 levels and macrophage content (NSD) (spindle: r = 0.62, *p* = 0.003; round: r = 0.59, *p* = 0.005) (Figure 5a). Moreover, borderline positive correlations were observed between HO-1 levels and max lipid arc (spindle: r = 0.34, *p* = 0.06; round: r = 0.43, *p* = 0.02) (Figure 5b).

## 4. Discussion

In the present study we demonstrate higher HO-1 protein levels and the activation of its related Nrf2/HO-1 pathway in MDMs obtained from CAD patients as compared to those obtained from healthy subjects. For the first time, we analyzed this pathway in the different MDM morphotypes. Moreover, we show a positive association between HO-1 levels in MDMs obtained in vitro and the vulnerable coronary plaque features, as detected in vivo by OCT.

HO-1 is a stress response protein that is expressed in several cell types, including macrophages. It is induced by several stimuli inducing oxidative stress, such as cardiovascular risks factors [13,14,15], hypoxia [40], and GSH depletion [41]. In line with in animal models studies [42,43], our previous data obtained in whole blood from CAD patients demonstrated a reduction of GSH levels and a related imbalance of the GSH/GSSG ratio, that is commonly used as an index of oxidative status [44,45]. Here, we detect a progressive decrease of this marker in MDMs obtained from SA, NSTEMI, and STEMI patients, indicating an increase of oxidative stress status in patients with a worse prognosis.

Augmented oxidative stress levels activate the Nrf2/HO-1 pathway as one of the cellular protective mechanisms. The important protective role of HO-1 against human atherosclerosis has been highlighted in population genetic studies that evidenced a polymorphism in the promoter region of human HO-1 gene associated with atherosclerosis predisposition [46,47,48]. In the clinical setting HO-1 deficiency is a very rare condition, but the autopsy case report of HO-1 deficient boy showed hyperlipidemia, presence of foam cells in the liver, and of fatty streak and fibrous plaque in the aorta. These are several lines of evidence that outline the role of HO-1 against atherogenesis [49].

Nevertheless, in our experimental conditions, MDMs from CAD patients expressed a progressive increase of HO-1 levels in both morphotypes, going from healthy to STEMI patients. This is in line with previous studies that infer an adaptive defense role of HO-1 against atherosclerosis [50]. The mechanisms underlying the cardioprotective function of HO-1 involve the antioxidant action of its products [51,52], the reduction in leukocyte recruitment and infiltration, and the suppression of the pro-inflammatory response of immune cells [18].

The increase in HO-1 levels observed in our study CAD population goes in parallel with the increased levels of Nrf2, the transcription factor involved in cell protection from oxidant stressors. Several results strengthen the anti-atherogenic role of Nrf2 in preserving vascular integrity and endothelial function [53], potentially through the release of NO [54] and the protection from cell apoptosis [55]. Moreover, the activation of Nrf2 protects human coronary artery endothelial cells against oxidative challenge [56]. In the present study, we also demonstrate the activation of Nrf2 pathway by its translocation into the nucleus. Indeed, CAD MDMs show higher levels in the nucleus as compared to healthy MDMs. In turn, the latter exhibit very low and feeble protein levels in the nucleus, and high protein levels in the cytosol. This is in line with Nrf2 cytosolic localization in basal condition, where Nrf2 is associated to Keap-1 protein, which induces its ubiquitination and proteasomal degradation. The increase of the Nrf2 stability and its translocation into the nucleus in response to stress signals activates the antioxidant gene transcription [57].

This defense mechanism is evidenced also in atherosclerotic plaques by the presence of high levels of HO-1. Notably, its levels in both carotid [16] and coronary [58] lesions positively correlated with atherosclerotic grade and plaque vulnerability. In addition, Li et al. showed increased levels of HO-1 in lymphocytes and monocytes associated with the severity of the pathology [59]. Accordingly, in our study, patients with the highest levels of HO-1 in MDMs, more frequently showed a vulnerable coronary plaque featured by a TCFA, and an increase in macrophage and lipid content. In addition, in our study, higher levels of HO-1 are detected in MDMs of CAD patients that presented with ruptured plaque and with the presence of thrombus as compared to those with non-ruptured plaque and without thrombi formation. This finding may be explained in light of previous studies reporting a positive correlation between HO-1 and matrix metalloproteinase-9 levels and a negative correlation between the presence of smooth muscle cells and collagen deposition [16]. Moreover, it has been observed that MMP-9 is abundant in carotid plaques characterized by the presence of intraplaque hemorrhage [60].

Overall, these results highlight the activation of Nrf2/HO-1 pathway as an antioxidant response mechanism in MDMs from CAD patients and point out that HO-1 levels may reflect coronary plaque vulnerability. This association could help in identifying patients with rupture-prone plaque and suggests Nrf2/HO-1 pathway as a new potential therapeutic target to counteract plaque progression.

## Figures and Tables

**Table 1:** Baseline clinical, laboratory, and angiographic characteristics of the study subjects.

Variables	Healthy Subjects (*n* = 10)	CAD (*n* = 30)	*p* Value Healthy Subjects vs. CAD °°	CAD			
				SA (*n* = 10)	NSTEMI (*n* = 10)	STEMI (*n* = 10)	ANOVA *p* Value °
*Demographics*							
Age (years)	61.5 ± 10	63.8 ± 12.1	0.5927	70.3 ± 7.2	61.0 ± 11.9	60.0 ± 14.8	0.1660
Male sex, n (%)	5 (50)	26 (86.7)	**0.0290** ^‡^	8 (80)	9 (90)	8 (80)	0.8179 ^‡^
Body mass index (kg/m^2^)	23.5 ± 1.6	29.3 ± 4.6	**0.0004**	28.0 ± 4.5 *	28.1 ± 3.7 *	32.3 ± 4.6 *	**0.0002**
*Clinical characteristics*							
Current smoking, n (%)	0	18 (60)	**0.0010** ^‡^	7 (70)	6 (60)	5 (50)	0.5884 ^‡^
Diabetes mellitus, n (%)	0	16 (53.3)	**0.0030** ^‡^	5 (50)	5 (50)	6 (60)	0.6593 ^‡^
Dyslipidemia, n (%)	0	16 (53.3)	**0.0030** ^‡^	7 (70)	5 (50)	4 (40)	0.4704 ^‡^
Hypertension, n (%)	0	14 (46.7)	**0.0070** ^‡^	4 (40)	5 (50)	5 (50)	0.9004 ^‡^
Family history of CAD, n (%)	0	17 (56.7)	**0.0020** ^‡^	4 (40)	9 (90) ^#^	4 (40)	**0.0149** ^‡^
LVEF (%)	NA	50.1 ± 8.8		48 ± 9.3	51.3 ± 9.8	51.0 ± 7.7	0.7510
*Laboratory data*							
WBC (× 10^9^/L)	7.6 ± 2.9	9.2 ± 3.9	0.2537	8.9 ± 2.4	9.1 ± 5.1	9.7 ± 4.0	0.6852
RBC (× 10^12^/L)	4.5 ± 0.8	5.1 ± 2.0	0.3917	4.6 ± 0.6	5.1 ± 0.6	5.8 ± 3.6	0.4330
Neutrophil count (× 10^9^/L)	4.8 ± 2.3	6.2 ± 3.5	0.2585	5.8 ± 2.3	6.1 ± 4.7	6.9 ± 3.4	0.6249
Lymphocyte count (× 10^9^/L)	2.1 ± 1.2	2.0 ± 0.9	0.7190	2.3 ± 1.2	1.9 ± 0.7	1.8 ± 0.9	0.6889
Eosinophil count (× 10^9^/L)	0.1 ± 0.1	0.2 ± 0.2	0.6303	0.2 ± 0.1	0.1 ± 0.1	0.2 ± 0.4	0.6123
Monocyte count (× 10^9^/L)	0.5 ± 0.2	0.6 ± 0.3	0.1398	0.6 ± 0.2	0.6 ± 0.4	0.7 ± 0.4	0.3176
Basophil count (× 10^9^/L)	0.03 ± 0.02	0.01 ± 0.00	**0.0072**	0.01 ± 0.02	0.01 ± 0.03 *	0.01 ± 0.02	**0.0343**
Platelets (× 10^9^/L)	248 ± 61.9	230.8 ± 83.4	0.5714	213.7 ± 49.8	252.2 ± 106.0	223.7 ± 85.6	0.6549
hs-CRP (mg/L)	1.9 (1.4–2.3)	4.9 (2.0–21.0)	**0.0141** ^†^	2.1 (1.6–2.1) ^ǂ #^	6.7 (1.6–17.0) *	38.6 (6.0–75.0) *	**0.0003** ^§^
Creatinine (mg/dL)	1 ± 0.1	1.0 ± 0.5	0.9372	0.8 ± 0.3 ^#^	0.8 ± 0.3 ^#^	1.4 ± 0.5	**0.0015**
Glycaemia (mg/dL)	93.5 ± 12.2	140.2 ± 42.8	**0.0017**	116.4 ± 27.1 ^#^	130.3 ± 33.4 ^#^	178.8 ± 43.7 *	<**0.0001**
Total cholesterol (mg/dL)	187.7 ± 22.1	204.4 ± 42.6	0.2438	181.1 ± 34.1 ^#^	207.9 ± 42.8	226.1 ± 41.7	**0.0417**
LDL (mg/dL)	112.6 ± 26	122.4 ± 41.9	0.4924	102.0 ± 23.5	130.4 ± 47.5	135.2 ± 46.2	0.1890
HDL (mg/dL)	41.1 ± 5.3	48.83 ± 14.9	**0.0242**	61.6 ± 16.7 *^,ǂ,#^	44.5 ± 10.5	43.3 ± 9.3	**0.0004**
Triglycerides (mg/dL)	143.8 ± 31.6	161.2 ± 55.5	0.3333	117.9 ± 42.7 ^ǂ#^	176.7 ± 59.6	190.3 ± 32.2	**0.0034**
Peak TnI (μg/dL)	NA	1 (0.0–29.4)		NA	1.2 (0.5–1.4) ^#^	29.7 (25.0–163.0)	<**0.0001** ^§^
Peak CK-MB (μg/dL)	NA	11.1 (2.1–110.0)		2.1 (1.5–2.1)	12.3 (2.5–28.0) ^#^	281 (110.0–521.0) ^#^	<**0.0001** ^§^
*Angiographic data*							
Culprit or treated vessel, n (%)							0.1489 ^‡^
LAD	NA	14 (46.7)		3 (30)	8 (80)	3 (30)	
LCX	NA	10 (30.3)		4 (40)	1 (10)	5 (50)	
RCA	NA	6 (20)		3 (30)	1 (10)	2 (20)	
Multivessel disease, n (%)	NA	17 (56.7)		8 (80)	4 (40)	5 (50)	0.3276 ^‡^
*Admission therapy*							
ASA, n (%)	0	11 (36.7)	**0.0380** ^‡^	3 (30)	5 (50)	3 (30)	0.3192 ^‡^
Beta-Blockers, n (%)	0	8 (26.7)	0.1650 ^‡^	2 (20)	5 (50)	1 (10)	0.2319 ^‡^
ACE-inhibitors, n (%)	0	9 (30)	0.0810 ^‡^	5 (50)	2 (20)	2 (20)	0.3192 ^‡^
Statins, n (%)	0	10 (30.3)	**0.0430** ^‡^	5 (50)	2 (20)	3 (30)	0.3459 ^‡^

SA: Stable angina; CAD: Coronary artery disease; LVEF: Left ventricular ejection fraction; WBC: White blood cells; RBC: Red blood cells; LDL: Low-density lipoprotein; HDL: High-density lipoprotein; hs-CRP: High-sensitive C-reactive protein: TnI: Troponin-I; CK-MB: Creatine phosphokinase-MB; LAD: Left anterior descending; LCX: Left circumflex; RCA: Right coronary artery; ASA: Aspirin; ACE-inhibitors, angiotensin-converting enzyme-inhibitors; NA: Not assessed. Data are expressed as mean ± SD or median and interquartile range. * *p* < 0.05 vs. healthy subjects; ǂ *p* < 0.05 vs. NSTEMI; # *p* < 0.05 vs. STEMI; ° by ANOVA test, except: § Kruskal–Wallis Test, ‡ Fisher exact test; °° by Independent t-test, except: † Wilcoxon rank-sum test, ‡ Fisher exact test.

**Table 2:** Optical coherence tomography features of coronary plaque in CAD patients.

Variables	CAD (*n* = 30)
Lipid plaque, *n* (%)	26 (86.67)
Fibrous plaque, *n* (%)	1 (3.33)
Calcific plaque, *n* (%)	3 (10)
Plaque rupture, *n* (%)	15 (50)
MLA, mm^2^ (IQR)	1.70 (1.43–2.58)
TCFA, *n* (%)	15 (50)
Thrombus, *n* (%)	14 (46.67)
Lipid quadrants, *n*	2.70 ± 1.02
Lipid arc, degree ° (IQR)	163 (133.5–280)
Max lipid arc, degree °	206.37 ± 87.10
Macrophage infiltration detection, *n* (%)	21 (70)
Macrophage NSD	6.24 ± 1.16

MLA: Minimal lumen area; TCFA: Thin-cap fibroatheroma; NSD: Normalized standard deviation. Data are expressed as mean ± SD or median and IQR.
